# [μ-1,2-Bis(diphenyl­phosphan­yl)-1,2-diethyl­hydrazine-κ^2^ *P*:*P*′]bis­[chlorido­gold(I)] tetra­hydro­furan disolvate

**DOI:** 10.1107/S1600536811000109

**Published:** 2011-01-12

**Authors:** Frederik H. Kriel, Manuel A. Fernandes, Judy Coates

**Affiliations:** a Project AuTEK, Mintek, Private Bag X3015, Randburg 2125, South Africa; b Molecular Science Institute, School of Chemistry, University of the Witwatersrand, PO Wits, 2050 Johannesburg, South Africa

## Abstract

The title compound, [Au_2_Cl_2_(C_28_H_30_N_2_P_2_)]·2C_4_H_8_O, was synthesized from a bidentate phosphine ligand complexed to two linear gold(I) chloride moieties. The Au(I) atom is in an almost linear coordination with a P—Au—Cl angle of 179.22 (4)°. The complex molecules reside on a twofold rotation axis.

## Related literature

For the structure of the parent ligand, see: Kriel *et al.* (2010*a* ▶). For the synthesis of the parent ligand and related structures utilizing alternative metals, see; Reddy *et al.* (1994 ▶, 1995 ▶); Slawin *et al.* (2002 ▶); Kriel *et al.* (2010*b* ▶, 2011 ▶). For Au⋯Au inter­actions, see: Holleman & Wiberg (2001 ▶). 
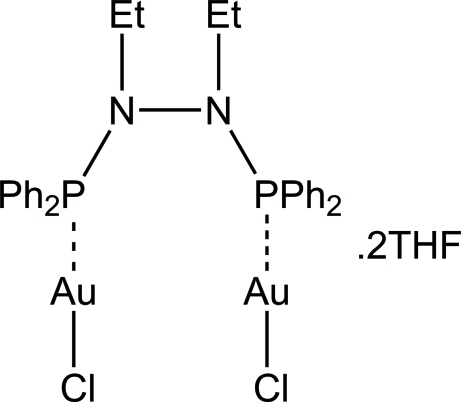


## Experimental

### 

#### Crystal data

[Au_2_Cl_2_(C_28_H_30_N_2_P_2_)]·2C_4_H_8_O
*M* *_r_* = 1065.52
Orthorhombic, 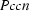

*a* = 12.3275 (18) Å
*b* = 17.200 (3) Å
*c* = 18.173 (3) Å
*V* = 3853.4 (10) Å^3^
*Z* = 4
Mo *K*α radiation
μ = 7.86 mm^−1^
*T* = 173 K
0.48 × 0.23 × 0.14 mm

#### Data collection

Bruker SMART CCD area-detector diffractometer
Absorption correction: integration (*SADABS*; Bruker, 1999 ▶) *T*_min_ = 0.116, *T*_max_ = 0.406
23024 measured reflections
4199 independent reflections
3256 reflections with *I* > 2σ(*I*)
*R*_int_ = 0.045

#### Refinement

*R*[*F* ^2^ > 2σ(*F* ^2^)] = 0.026
*wR*(*F* ^2^) = 0.052
*S* = 1.06
4199 reflections
209 parameters
H-atom parameters constrained
Δρ_max_ = 0.52 e Å^−3^
Δρ_min_ = −1.22 e Å^−3^

### 

Data collection: *SMART-NT* (Bruker, 1998 ▶); cell refinement: *SAINT-Plus* (Bruker, 1999 ▶); data reduction: *SAINT-Plus*; program(s) used to solve structure: *SHELXS97* (Sheldrick, 2008 ▶); program(s) used to refine structure: *SHELXL97* (Sheldrick, 2008 ▶); molecular graphics: *ORTEP-3* (Farrugia, 1997 ▶) and *Mercury* (Macrae *et al.*, 2008) ▶; software used to prepare material for publication: *WinGX* (Farrugia, 1999 ▶).

## Supplementary Material

Crystal structure: contains datablocks I, global. DOI: 10.1107/S1600536811000109/pb2050sup1.cif

Structure factors: contains datablocks I. DOI: 10.1107/S1600536811000109/pb2050Isup2.hkl

Additional supplementary materials:  crystallographic information; 3D view; checkCIF report

## Figures and Tables

**Fig. 1.**
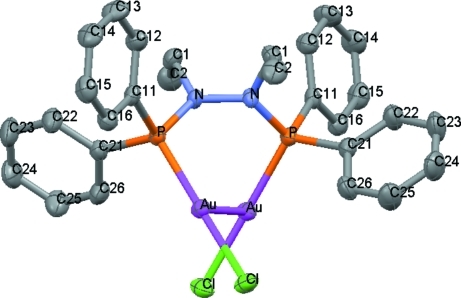
Molecular structure drawn with displacement ellipsoids at the 50% probability level. Hydrogen atoms and solvent THF have been omitted for clarity.

**Fig. 2.**
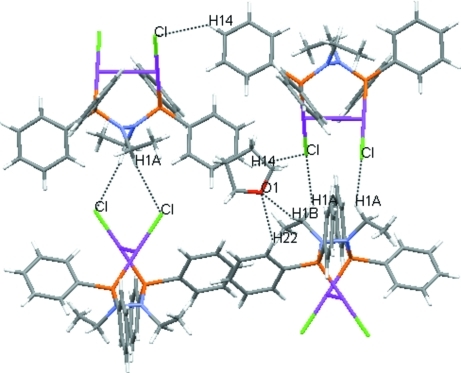
Packing of the title compound showing short contacts.

## supplementary crystallographic information

### Comment

Gold(I) forms an almost linear complex with a P—Au—Cl angles of 179.22 (4) °. The Au—Au distance of the complex is 3.131 (5) Å and is intramolecular. This is well within the range of aurophilic interactions as discribed by Holleman *et al.* as being normally between 2.7 Å and 3.4 Å. Other bond lengths are within expected ranges.

The molecule readilly crystalizes out of tetrahydrofuran (THF) and also includes this solvent in the crystal lattice. The molecule exhibits columns of head-to-tail complexes forming channels filled with THF. Intermolecular short contacts of 2.822 Å and 2.835 Å are displayed between Cl and H(1a) and Cl and H(14), respectively (Figure 2). The Cl—H(1a) interaction can be seen on the head-to-tail arranged complexes in a column between chlorines and adjacent hydrogen atoms situated on the ethyl substituted hydrazine bridge. The Cl—H(14) interaction occurs between chlorines and H atoms situated on a phenyl ring in close proximity. Hydrogen bonding distances between the THF O(1) atom and H(1 b) and H(22) are observed to be 2.553 Å and 2.698 Å, respectively.

### Experimental

Tetrahydrothiophenegold(I) chloride [(THT)AuCl] was suspended in THF. 0.5 equivalents of the ligand bis(diphenylphosphino)-1,2-diethylhydrazine dissolved in dichloromethane (DCM) was added to the stirred suspension. The suspension turned yellow and became clear and after a short time micro crystals started to form. Crystals of the molecule was obtained by halting stirring as soon as the reaction turned clear, crystals big enough for single-crystal X-Ray analysis formed overnight.

### Refinement

The H atoms were positioned geometrically and allowed to ride on their respective parent atoms, with C—H = 0.93 (Ar—H) or 0.96 (CH_3_) Å, and with *U*_eq_ = 1.2 (Ar—H) or 1.5 (CH_3_) *U*_eq_(C).

### Crystal data

**Table:**

[Au_2_Cl_2_(C_28_H_30_N_2_P_2_)]·2C_4_H_8_O	*F*(000) = 2056
*M**_r_* = 1065.52	*D*_x_ = 1.837 Mg m^−^^3^
Orthorhombic, *P**c**c**n*	Mo *K*α radiation, λ = 0.71073 Å
Hall symbol: -P 2ab 2ac	Cell parameters from 8545 reflections
*a* = 12.3275 (18) Å	θ = 6.1–55.9°
*b* = 17.200 (3) Å	µ = 7.86 mm^−^^1^
*c* = 18.173 (3) Å	*T* = 173 K
*V* = 3853.4 (10) Å^3^	Prismic, colourless
*Z* = 4	0.48 × 0.23 × 0.14 mm

### Data collection

**Table:**

Bruker SMART CCD area-detector diffractometer	4199 independent reflections
Radiation source: fine-focus sealed tube	3256 reflections with *I* > 2σ(*I*)
graphite	*R*_int_ = 0.045
φ and ω scans	θ_max_ = 27.0°, θ_min_ = 2.0°
Absorption correction: integration (*SADABS*; Bruker, 1999)	*h* = −11→15
*T*_min_ = 0.116, *T*_max_ = 0.406	*k* = −21→21
23024 measured reflections	*l* = −22→23

### Refinement

**Table:**

Refinement on *F*^2^	Primary atom site location: structure-invariant direct methods
Least-squares matrix: full	Secondary atom site location: difference Fourier map
*R*[*F*^2^ > 2σ(*F*^2^)] = 0.026	Hydrogen site location: inferred from neighbouring sites
*wR*(*F*^2^) = 0.052	H-atom parameters constrained
*S* = 1.06	*w* = 1/[σ^2^(*F*_o_^2^) + (0.0196*P*)^2^ + 3.5032*P*] where *P* = (*F*_o_^2^ + 2*F*_c_^2^)/3
4199 reflections	(Δ/σ)_max_ = 0.002
209 parameters	Δρ_max_ = 0.52 e Å^−^^3^
0 restraints	Δρ_min_ = −1.22 e Å^−^^3^

### Special details

**Table:**

Experimental. Reaction: bis(diphenylphosphino)-1,2-diethylhydrazine: 155 mg (0.34 mmol), (THT)AuCl: 200 mg (0.68 mmol), tetrahydrofuran: 2 ml, dichloromethane: 5 ml, Yield: 86%. Colourless to grey crystals. ^1^H NMR: (d-DMSO, 300 MHz) δH 7.90 (dq, Arom, J (^1^H-^31^P) = 28.9, J (^1^H-^1^H) = 7.1 Hz), 7.71 (d, Arom, J = 7.0 Hz), 7.60 (d, Arom, J = 7.1 Hz), 7.55 (d, Arom, J (^1^H-^1^H) = 7.0 Hz), 3.33 (bs, CH2CH3), 0.43 (t, CH2CH3, ^3^J (^1^H-^1^H) = 6.6 Hz). ^13^C NMR:(d-DMSO, 75 MHz) δC 132.3 (bs, Arom), 130.6 (m, Arom), 129.0 (s, Arom), 128.0 (bs, Arom), 43.6 (bs, *CH_2_*CH_3_), 14.1 (d, CH_2_*CH_3_*, ^3^J (^13^C-^31^P) = 16.1 Hz). ^31^P NMR:(d-DMSO, 121 MHz) δP 87.7. MS: 920 (2%, *M*), 885 (14%, *M* - Cl). EA: Calc: (Au_2_Cl_2_P_2_N_2_C_28_H_30_) C 36.50%, H 3.28%, N 3.04%, Found: C 35.42%, H 3.44%, N 2.64%. MP: 202 - 204 °C.
Geometry. All e.s.d.'s (except the e.s.d. in the dihedral angle between two l.s. planes) are estimated using the full covariance matrix. The cell e.s.d.'s are taken into account individually in the estimation of e.s.d.'s in distances, angles and torsion angles; correlations between e.s.d.'s in cell parameters are only used when they are defined by crystal symmetry. An approximate (isotropic) treatment of cell e.s.d.'s is used for estimating e.s.d.'s involving l.s. planes.
Refinement. Refinement of *F*^2^ against ALL reflections. The weighted *R*-factor *wR* and goodness of fit *S* are based on *F*^2^, conventional *R*-factors *R* are based on *F*, with *F* set to zero for negative *F*^2^. The threshold expression of *F*^2^ > σ(*F*^2^) is used only for calculating *R*-factors(gt) *etc*. and is not relevant to the choice of reflections for refinement. *R*-factors based on *F*^2^ are statistically about twice as large as those based on *F*, and *R*- factors based on ALL data will be even larger.

### Fractional atomic coordinates and isotropic or equivalent isotropic displacement parameters (Å^2^)

**Table:**

	*x*	*y*	*z*	*U*_iso_*/*U*_eq_	
Au	0.816153 (12)	0.172310 (9)	1.064560 (8)	0.02482 (5)	
Cl	0.73414 (9)	0.12548 (7)	1.16939 (5)	0.0353 (3)	
N	0.8044 (2)	0.26317 (18)	0.90903 (17)	0.0226 (7)	
P	0.89483 (8)	0.21634 (6)	0.96202 (6)	0.0226 (2)	
C1	0.8360 (3)	0.3202 (2)	0.8518 (2)	0.0300 (10)	
H1A	0.7918	0.3109	0.8071	0.036*	
H1B	0.9130	0.3117	0.8386	0.036*	
C2	0.8210 (4)	0.4044 (2)	0.8762 (3)	0.0413 (11)	
H2A	0.7458	0.4125	0.8917	0.062*	
H2B	0.8379	0.4392	0.8350	0.062*	
H2C	0.8699	0.4155	0.9174	0.062*	
C11	0.9634 (3)	0.1431 (2)	0.9067 (2)	0.0244 (9)	
C12	0.9538 (3)	0.1404 (3)	0.8308 (2)	0.0347 (10)	
H12	0.9089	0.1768	0.8060	0.042*	
C13	1.0099 (4)	0.0845 (3)	0.7910 (3)	0.0428 (12)	
H13	1.0027	0.0826	0.7390	0.051*	
C14	1.0760 (4)	0.0317 (3)	0.8262 (3)	0.0398 (11)	
H14	1.1155	−0.0055	0.7984	0.048*	
C15	1.0848 (4)	0.0329 (3)	0.9009 (3)	0.0393 (11)	
H15	1.1297	−0.0041	0.9250	0.047*	
C16	1.0283 (3)	0.0879 (2)	0.9421 (2)	0.0301 (9)	
H16	1.0339	0.0880	0.9942	0.036*	
C21	0.9994 (3)	0.2878 (2)	0.9796 (2)	0.0267 (9)	
C22	1.0943 (3)	0.2924 (3)	0.9375 (3)	0.0402 (11)	
H22	1.1069	0.2561	0.8991	0.048*	
C23	1.1695 (4)	0.3501 (3)	0.9521 (3)	0.0532 (15)	
H23	1.2336	0.3534	0.9232	0.064*	
C24	1.1521 (4)	0.4029 (3)	1.0083 (3)	0.0515 (14)	
H24	1.2043	0.4423	1.0178	0.062*	
C25	1.0602 (4)	0.3988 (3)	1.0502 (3)	0.0471 (13)	
H25	1.0482	0.4353	1.0885	0.057*	
C26	0.9856 (4)	0.3416 (3)	1.0367 (3)	0.0390 (11)	
H26	0.9228	0.3382	1.0668	0.047*	
O1	0.0875 (5)	0.3175 (4)	0.7631 (3)	0.139 (3)	
C31	0.1485 (8)	0.3817 (6)	0.7578 (4)	0.134 (4)	
H31A	0.2149	0.3755	0.7879	0.161*	
H31B	0.1076	0.4269	0.7771	0.161*	
C32	0.1791 (6)	0.3963 (4)	0.6793 (4)	0.093 (2)	
H32A	0.1566	0.4488	0.6632	0.111*	
H32B	0.2582	0.3904	0.6718	0.111*	
C33	0.1174 (7)	0.3346 (4)	0.6396 (4)	0.099 (3)	
H33A	0.0610	0.3582	0.6078	0.119*	
H33B	0.1667	0.3033	0.6084	0.119*	
C34	0.0669 (6)	0.2854 (5)	0.6968 (4)	0.095 (2)	
H34A	−0.0124	0.2821	0.6887	0.114*	
H34B	0.0973	0.2321	0.6947	0.114*	

### Atomic displacement parameters (Å^2^)

**Table:**

	*U*^11^	*U*^22^	*U*^33^	*U*^12^	*U*^13^	*U*^23^
Au	0.02502 (8)	0.02674 (8)	0.02271 (8)	0.00606 (7)	0.00177 (7)	0.00268 (7)
Cl	0.0369 (6)	0.0436 (6)	0.0253 (5)	0.0084 (5)	0.0066 (5)	0.0081 (5)
N	0.0158 (16)	0.0250 (17)	0.0270 (16)	0.0007 (14)	0.0011 (13)	0.0051 (14)
P	0.0190 (5)	0.0254 (5)	0.0234 (5)	0.0038 (4)	−0.0002 (4)	0.0010 (4)
C1	0.026 (2)	0.034 (2)	0.031 (2)	0.0036 (19)	0.0055 (17)	0.0080 (19)
C2	0.046 (3)	0.031 (2)	0.046 (3)	−0.002 (2)	−0.002 (2)	0.011 (2)
C11	0.018 (2)	0.024 (2)	0.030 (2)	0.0037 (16)	0.0022 (17)	0.0007 (17)
C12	0.032 (2)	0.040 (2)	0.032 (2)	0.009 (2)	0.001 (2)	0.003 (2)
C13	0.050 (3)	0.047 (3)	0.032 (3)	0.012 (2)	0.009 (2)	−0.010 (2)
C14	0.040 (3)	0.038 (3)	0.041 (3)	0.014 (2)	0.006 (2)	−0.009 (2)
C15	0.032 (3)	0.035 (3)	0.051 (3)	0.010 (2)	−0.003 (2)	0.000 (2)
C16	0.028 (2)	0.030 (2)	0.032 (2)	0.0044 (18)	−0.0014 (19)	−0.0013 (19)
C21	0.021 (2)	0.030 (2)	0.029 (2)	0.0029 (18)	−0.0009 (17)	0.0011 (19)
C22	0.029 (2)	0.047 (3)	0.044 (3)	−0.003 (2)	0.005 (2)	−0.011 (2)
C23	0.028 (3)	0.070 (4)	0.062 (4)	−0.016 (2)	0.009 (2)	−0.013 (3)
C24	0.039 (3)	0.056 (3)	0.059 (3)	−0.023 (3)	−0.002 (2)	−0.009 (3)
C25	0.042 (3)	0.048 (3)	0.051 (3)	−0.007 (2)	0.004 (2)	−0.019 (2)
C26	0.030 (2)	0.047 (3)	0.040 (3)	−0.006 (2)	0.009 (2)	−0.001 (2)
O1	0.142 (5)	0.228 (7)	0.048 (3)	−0.110 (5)	0.010 (3)	−0.001 (4)
C31	0.184 (10)	0.148 (9)	0.071 (6)	−0.072 (8)	0.018 (6)	−0.011 (6)
C32	0.090 (5)	0.092 (5)	0.096 (6)	−0.025 (4)	0.018 (5)	0.016 (5)
C33	0.122 (6)	0.125 (7)	0.050 (4)	−0.060 (5)	−0.016 (4)	0.019 (4)
C34	0.095 (5)	0.131 (6)	0.059 (4)	−0.035 (5)	−0.005 (4)	0.020 (4)

### Geometric parameters (Å, °)

**Table:**

Au—P	2.2331 (11)	C21—C22	1.399 (6)
Au—Cl	2.3021 (10)	C21—C26	1.400 (6)
Au—Au^i^	3.1310 (5)	C22—C23	1.383 (6)
N—N^i^	1.416 (6)	C22—H22	0.9500
N—C1	1.481 (5)	C23—C24	1.385 (7)
N—P	1.679 (3)	C23—H23	0.9500
P—C21	1.810 (4)	C24—C25	1.367 (6)
P—C11	1.820 (4)	C24—H24	0.9500
C1—C2	1.526 (6)	C25—C26	1.369 (6)
C1—H1A	0.9900	C25—H25	0.9500
C1—H1B	0.9900	C26—H26	0.9500
C2—H2A	0.9800	O1—C31	1.340 (9)
C2—H2B	0.9800	O1—C34	1.349 (8)
C2—H2C	0.9800	C31—C32	1.497 (9)
C11—C12	1.386 (6)	C31—H31A	0.9900
C11—C16	1.397 (5)	C31—H31B	0.9900
C12—C13	1.388 (6)	C32—C33	1.491 (9)
C12—H12	0.9500	C32—H32A	0.9900
C13—C14	1.377 (6)	C32—H32B	0.9900
C13—H13	0.9500	C33—C34	1.479 (8)
C14—C15	1.361 (6)	C33—H33A	0.9900
C14—H14	0.9500	C33—H33B	0.9900
C15—C16	1.394 (6)	C34—H34A	0.9900
C15—H15	0.9500	C34—H34B	0.9900
C16—H16	0.9500		
			
P—Au—Cl	179.22 (4)	C22—C21—P	122.5 (3)
P—Au—Au^i^	86.37 (3)	C26—C21—P	119.5 (3)
Cl—Au—Au^i^	94.01 (3)	C23—C22—C21	119.7 (4)
N^i^—N—C1	117.4 (3)	C23—C22—H22	120.1
N^i^—N—P	118.4 (3)	C21—C22—H22	120.1
C1—N—P	123.1 (2)	C22—C23—C24	120.6 (5)
N—P—C21	104.37 (17)	C22—C23—H23	119.7
N—P—C11	108.89 (18)	C24—C23—H23	119.7
C21—P—C11	103.71 (18)	C25—C24—C23	120.3 (5)
N—P—Au	110.68 (11)	C25—C24—H24	119.8
C21—P—Au	113.12 (13)	C23—C24—H24	119.8
C11—P—Au	115.30 (14)	C24—C25—C26	119.6 (5)
N—C1—C2	113.1 (3)	C24—C25—H25	120.2
N—C1—H1A	109.0	C26—C25—H25	120.2
C2—C1—H1A	109.0	C25—C26—C21	121.7 (4)
N—C1—H1B	109.0	C25—C26—H26	119.1
C2—C1—H1B	109.0	C21—C26—H26	119.1
H1A—C1—H1B	107.8	C31—O1—C34	112.3 (6)
C1—C2—H2A	109.5	O1—C31—C32	110.3 (7)
C1—C2—H2B	109.5	O1—C31—H31A	109.6
H2A—C2—H2B	109.5	C32—C31—H31A	109.6
C1—C2—H2C	109.5	O1—C31—H31B	109.6
H2A—C2—H2C	109.5	C32—C31—H31B	109.6
H2B—C2—H2C	109.5	H31A—C31—H31B	108.1
C12—C11—C16	118.9 (4)	C33—C32—C31	102.4 (6)
C12—C11—P	122.2 (3)	C33—C32—H32A	111.3
C16—C11—P	118.8 (3)	C31—C32—H32A	111.3
C11—C12—C13	120.0 (4)	C33—C32—H32B	111.3
C11—C12—H12	120.0	C31—C32—H32B	111.3
C13—C12—H12	120.0	H32A—C32—H32B	109.2
C14—C13—C12	120.6 (4)	C34—C33—C32	106.3 (6)
C14—C13—H13	119.7	C34—C33—H33A	110.5
C12—C13—H13	119.7	C32—C33—H33A	110.5
C15—C14—C13	120.0 (4)	C34—C33—H33B	110.5
C15—C14—H14	120.0	C32—C33—H33B	110.5
C13—C14—H14	120.0	H33A—C33—H33B	108.7
C14—C15—C16	120.4 (4)	O1—C34—C33	108.3 (6)
C14—C15—H15	119.8	O1—C34—H34A	110.0
C16—C15—H15	119.8	C33—C34—H34A	110.0
C15—C16—C11	120.0 (4)	O1—C34—H34B	110.0
C15—C16—H16	120.0	C33—C34—H34B	110.0
C11—C16—H16	120.0	H34A—C34—H34B	108.4
C22—C21—C26	118.0 (4)		
			
N^i^—N—P—C21	−155.3 (2)	C14—C15—C16—C11	−0.9 (7)
C1—N—P—C21	37.0 (3)	C12—C11—C16—C15	1.9 (6)
N^i^—N—P—C11	94.4 (3)	P—C11—C16—C15	−177.7 (3)
C1—N—P—C11	−73.3 (3)	N—P—C21—C22	−95.7 (4)
N^i^—N—P—Au	−33.3 (3)	C11—P—C21—C22	18.3 (4)
C1—N—P—Au	159.0 (3)	Au—P—C21—C22	144.0 (3)
Au^i^—Au—P—N	−38.27 (13)	N—P—C21—C26	83.6 (4)
Au^i^—Au—P—C21	78.44 (14)	C11—P—C21—C26	−162.4 (3)
Au^i^—Au—P—C11	−162.41 (14)	Au—P—C21—C26	−36.8 (4)
N^i^—N—C1—C2	90.6 (4)	C26—C21—C22—C23	−1.4 (7)
P—N—C1—C2	−101.6 (4)	P—C21—C22—C23	177.8 (4)
N—P—C11—C12	11.5 (4)	C21—C22—C23—C24	0.5 (8)
C21—P—C11—C12	−99.2 (4)	C22—C23—C24—C25	0.0 (9)
Au—P—C11—C12	136.5 (3)	C23—C24—C25—C26	0.5 (8)
N—P—C11—C16	−168.9 (3)	C24—C25—C26—C21	−1.6 (8)
C21—P—C11—C16	80.4 (3)	C22—C21—C26—C25	2.0 (7)
Au—P—C11—C16	−43.8 (4)	P—C21—C26—C25	−177.3 (4)
C16—C11—C12—C13	−1.2 (6)	C34—O1—C31—C32	−2.4 (12)
P—C11—C12—C13	178.4 (3)	O1—C31—C32—C33	5.2 (11)
C11—C12—C13—C14	−0.6 (7)	C31—C32—C33—C34	−5.8 (9)
C12—C13—C14—C15	1.6 (7)	C31—O1—C34—C33	−1.6 (11)
C13—C14—C15—C16	−0.8 (7)	C32—C33—C34—O1	4.9 (10)

Symmetry codes: (i) −*x*+3/2, −*y*+1/2, *z*.
